# The First Report of Ruminant Fascioliasis in Sabah, East Malaysia

**DOI:** 10.1155/2021/6691483

**Published:** 2021-04-17

**Authors:** Naim Che Kamaruddin, Muhammad Ali Imran Razali, Ibitoye Emmanuel Busayo, Noor Hazfalinda Hamzah, Lokman Hakim Idris, Nur Mahiza Md Isa

**Affiliations:** ^1^Department of Pathology and Microbiology, Faculty of Veterinary Medicine, Universiti Putra Malaysia, 43400 UPM Serdang Selangor, Malaysia; ^2^Department of Theriogenology and Animal Production, Faculty of Veterinary Medicine, Usmanu Danfodiyo University Sokoto, Nigeria; ^3^Forensic Science Program, Faculty of Health Sciences, Universiti Kebangsaan Malaysia, 43600 UKM Bangi, Selangor, Malaysia; ^4^Department of Veterinary Preclinical Sciences, Faculty of Veterinary Medicine, Universiti Putra Malaysia, 43400 UPM Serdang, Selangor, Malaysia

## Abstract

Ruminant fascioliasis is a neglected yet important tropical zoonotic disease that affects both the livestock and humans. The disease has a worldwide distribution, and Malaysia is one of the countries that face problems related to this parasite. These retrospective studies were conducted in Makmal Diagnosa Veterinar Kota Kinabalu (MDVKK) and Sabah Meat Technology Centre (SMTC), Kinarut over a period of eleven years (2008–2018). For MDVKK, the overall occurrence of fascioliasis was 24.9%. Out of 769 cattle's and buffaloes' faecal samples submitted, *Fasciola* spp ova were detected in 189 of the samples. A total of 2297 cattle, buffaloes, and goats were slaughtered at SMTC over that period, and 21 livers were condemned due to fascioliasis, giving the total occurrence of 0.91%. This investigation provides information on the occurrence of ruminant fascioliasis in Sabah, East Malaysia. The results from this study highlight the alarming incidence of fascioliasis and the urgent need for action to control this neglected tropical disease in East Malaysia.

## 1. Introduction

Fascioliasis is an important parasitic infection caused by two *Fasciola* spp, which are *Fasciola hepatica* and *Fasciola gigantica*. It is a neglected tropical disease that is capable of affecting both humans and livestock. World Health Organization [1] claimed that approximately 2.4 million people in more than 70 countries are estimated to be infected by fascioliasis. Occurrence ranged from 2.9% to 13.3% among 865 school children in Puebla State, Mexico, reflecting its zoonotic potential [2]. In Malaysia, the prevalence of ruminant fascioliasis has been reported from different parts of the country. Screening of slaughtered cattle in abattoir reported a prevalence of 7.5% from Ipoh, the northwest region of West Malaysia (WM) [3]. While in the East Coast region of WM, the first surveys in six large ruminant farms showed a ruminant fascioliasis of 67% [4]. While a retrospective study in WM involving analysis of a ten-year data from regional laboratories from main veterinary laboratories highlighted the northern part of WM is at higher risk [5]. Although *Fasciola* has been identified significantly in WM, no concerted efforts have been made to study this parasite in East Malaysia, despite Sabah being notified with human *Fasciola* in Tuaran [6]. Hence, the objective of this study is to determine the occurrence of fascioliasis in ruminants diagnosed at Makmal Diagnosa Veterinar Kota Kinabalu (MDVKK) and Sabah Meat Technology Centre (SMTC), Sabah to update current information on ruminant fascioliasis especially in East Malaysia. This will eventually stimulate the interest to intensify efforts on its monitoring and surveillance towards the control and possible eradication of this neglected disease in East Malaysia.

## 2. Materials and Methods

### 2.1. Sampling and Data Collection

The laboratory involved in this study is Makmal Diagnosa Veterinar Kota Kinabalu (MDVKK) which is situated at Kota Kinabalu Sabah. The retrospective data were collected from the laboratory, between January 2008 and December 2018. However, data from 2017 was not included in this study due to the fact that those raw data could not be retrieved. The laboratory database includes information on the source of sample, date of submission, and species of animals. Records were examined on an annual basis with regard to cases of fascioliasis reported in animals. The occurrence of fascioliasis was calculated as the proportion of positive samples out of the samples that were submitted. The proportion of samples that tested positive by year or host species or by districts was computed in a similar way.

**Figure 1:**
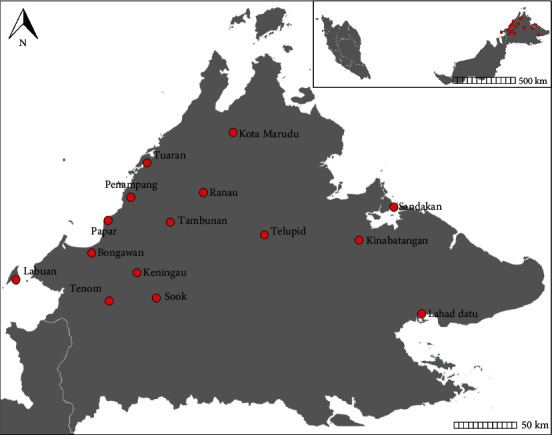
Smaller map shows Malaysia with emphasized on Sabah (containing red dots) and larger map zoomed the districts of Sabah (red dots). The samples from those districts were used in this study for diagnosing the occurrence of ruminant fascioliasis.

The study also involved the inspection of 11 years data from year 2008 to 2018 in Sabah Meat Technology Centre (SMTC). SMTC is one of the main slaughterhouses in Sabah as it receives animals from various districts (Figure 1). Upon postmortem inspection, livers were observed thoroughly for any abnormality. The condemnation of the liver was declared by the meat inspector based on the appearance of lesions suggestive of damaged tissues, adhesion, hemorrhage, or thickened bile duct. The presence of adult liver flukes indicated positive fascioliasis.

The significance of association between the occurrence of fascioliasis and year, host species and districts were evaluated using the chi-square test and quantified by computing the odds ratio. Data were entered, validated and calculated in Microsoft® Excel 2007 spreadsheet.

## 3. Result

### 3.1. The Occurrence of Ruminant Fascioliasis over 10- and 11-Year Period at MDVKK and SMTC

Table 1 illustrates the occurrence of fascioliasis at MDVKK and SMTC from 2008 to 2018. On the basis of the 10-year data from MDVKK (no data in year 2017), 194 were positive with *Fasciola* ova out of 986 samples, giving a total occurrence of 24.5% (*p* < 0.05). The highest occurrence was recorded in 2009, which was 48.4%, followed by 2011 and 2014 with the occurrences of 43.8% and 30.8%, respectively. Zero occurrence was recorded in 2013. Table 1 also shows the occurrence of fascioliasis over an 11-year period from SMTC. Based on the data from 2008 to 2018, a total of 2,297 large and small ruminants were slaughtered at SMTC. From the postmortem examination of the carcasses, 21 livers were condemned due to fascioliasis within this period, giving a total occurrence of 0.9% (*p* < 0.05). Year 2008 recorded the highest occurrence of fascioliasis, which was 8.4%, followed by year 2018 and 2011 with the occurrences of 1.1% and 0.6%, respectively. Zero occurrence was recorded in other years.

### 3.2. The Host-Specific Occurrence of Ruminant Fascioliasis over 10 and 11 Years Period at MDVKK and SMTC

Table 2 shows the host-specific occurrence of ruminant fascioliasis at MDVKK and SMTC. Based on the 10-year data from MDVKK, three different host species, cattle, buffalo, and goat, were diagnosed with fascioliasis. Cattle recorded the highest occurrence among all, with 31.4%, as compared to buffalo and goat with 8.7% and 2.3% occurrences, respectively. At SMTC, cattle had the highest occurrence of fascioliasis than buffaloes, which were 1.7% and 0.8%, respectively, and 0% occurrence of fascioliasis was observed in goats.

### 3.3. The District-Specific Occurrence of Ruminant Fascioliasis at Over 10 Years Period at MDVKK

The occurrence of ruminant fascioliasis according to districts is tabulated in Table 3. From 13 districts, Ranau recorded the highest occurrence of fascioliasis, which was 37.7%, followed by Tambunan, Sandakan, Papar, and Bongawan with the occurrences of 32.2%, 24.3%, 19.2%, and 16.7%, respectively (*p* < 0.05). The remaining districts recorded zero occurrence.

## 4. Discussion

This retrospective study provides information on ruminant fascioliasis in East Malaysia, Sabah. The result shows that the overall occurrence of ruminant fascioliasis at MDVKK and SMTC was 24.9% and 0.9%, respectively, over a 10- and 11-year period. The occurrence of fascioliasis was highest in cattle as compared to buffalo and goat. Ranau district reported the highest occurrence of fascioliasis compared with other 12 districts studied. The study has also revealed that the prevalence of ruminant fascioliasis at MDVKK is higher than at SMTC. This is in accordance with the fact that postmortem examination for detection of liver fluke may fail if the parasite burden is low. This is particularly the case at the early stage of infection when pathological changes in the liver due to fascioliasis is yet to be fully manifested, and, as a consequence, the meat inspector may misjudge a liver's condition. In addition, the immature liver fluke is not readily detectable by unaided human eyes.

The occurrence of fascioliasis among ruminants recorded in MDVKK was found to be higher as compared to the recent study conducted by Diyana et al. [5] in five main Veterinary Regional Laboratories in Peninsular Malaysia, which was 1.76% (35/1,988). The higher occurrence of fascioliasis can be attributed to the fact that Sabah climatically favours the survival of the intermediate host. According to [7], the average annual rainfall in Sabah and Peninsular Malaysia is 2,630 mm and 2,420 mm, respectively. Heavy rainfall provides a better environment for the breeding of *Lymnaea* spp, which is the intermediate host for *Fasciola* spp [8]. The overall occurrence of fascioliasis in ruminants slaughtered at SMTC was 0.91% (21/2,297) over the 11-year period. This is relatively low as compared to the occurrence of fascioliasis at abattoirs as such in North-central Nigeria (1.46%, 47,931/3,292,634) [9], Borno State, Nigeria (13.67%, 41/300) [10], and Sokoto, Nigeria (27.68%, 62/224) [11]. However, the occurrence at SMTC is higher to study in Botswana (0.09%, 1,250/1,400,000) [12]. The variability of occurrence between these studies is maybe associated with the differences in the study design as such sample size, host species, diagnostic method used for the infection status, and different husbandry management and systems [13].

The occurrence of fascioliasis was highest in cattle as compared to buffalo and goat in this research. The same finding where occurrence of fascioliasis in cattle was higher than other species was obtained by Ouchene-Khelifi et al. [14] at Algerian abattoirs and was supported with a recent study by Isah [15] with a similar finding in Bauchi state, Nigeria. The difference in occurrence is possibly the difference in grazing behavior among ruminants. Anatomically, bovines such as cattle and buffaloes have wider mouths and inflexible upper lips, which restrict the ability to select grasses, resulting in eating large clumps of grasses at one time. In contrast, small ruminants such as goats and sheep have narrower mouths and more flexible lips, which allow them to be more selective and graze less [16]. The anatomical features possessed by bovines cause a higher risk in ingesting metacercaria that are encysted on vegetation. Buffaloes had a lower occurrence of fascioliasis than cattle at both studied areas. This is in sharp contrast to earlier studies [17–19] which reported a higher infection rate among buffaloes than cattle. This is supported by the fact that buffaloes prefer swampy areas and like to wallow in muddy areas where intermediate hosts are abundant. The possible explanation could be that lesser number of buffaloes in this study; hence, the fascioliasis occurrence showed lesser in this study.

The general picture of ruminant fascioliasis in Sabah shows that the Ranau district was observed to have the highest occurrence of fascioliasis compared with other 12 districts under this investigation. Climate and the host density population might be the important factors in resulting the higher occurrence of fascioliasis in Ranau. This includes high mean annual rainfall (2,017 mm) and suitable temperature for *Fasciola* egg excreted by the ruminant hosts to mature and hatch into miracidia (14°C to 24°C) that will disperse the infective metacercariae cysts [20]. During the study period, ruminant fascioliasis was also high in Tambunan probably this district is surrounded by man-made agricultural irrigations such as paddy fields which provide an excellent environment for the proliferation of freshwater snails [20]. Madsen [21] supported the findings by stating that a high number of *Lymnaea* spp can be found in paddy fields in Vietnam. Since *Lymnaea* spp is the well-known intermediate host for fascioliasis, this may increase the chance of acquiring the infection. The abundance of water as irrigation for paddy fields provides a suitable environment for aquatic miracidia to swim and supports the life longevity of metacercaria encysted on water vegetations.

## 5. Conclusion

In conclusion, there is a significant occurrence of fascioliasis at MDVKK and SMTC, Sabah from 2008 until 2018. The occurrence at MDVKK is much higher compared to SMTC. The cattle recorded the highest occurrence as compared to buffalo and goat in the studied areas. Ranau, Tambunan, and Sandakan were noted for their higher fascioliasis occurrences compared to other districts in Sabah. The result from this study highlights the importance and urgent need of controlling this neglected disease.

## Figures and Tables

**Table 1:** The occurrence of ruminant fascioliasis at MDVKK and SMTC over 10- and 11-year period.

Years	MDVKK				SMTC			
	Total sample (N)	Total positive sample (*n*)	Occurrence of fascioliasis (%)	Confidence interval (95%)	Total animal slaughtered (*N*)	Total positive sample (*n*)	Occurrence of fascioliasis (%)	Confidence interval (95%)
2008	31	5	16.1	5.5–33.7	202	17	8.4	5.0–13.1
2009	258	125	48.4	42.4–54.7	183	0	0	0–2.0
2010	72	9	12.5	5.9–22.4	273	0	0	0–1.3
2011	32	14	43.8	26.4–62.3	177	1	0.6	0–3.1
2012	116	7	6.0	2.5–12.0	311	0	0	0–1.2
2013	26	0	0	0–13.2	155	0	0	0–2.4
2014	26	8	30.8	14.3–51.8	216	0	0	0–1.7
2015	46	4	8.7	2.4–20.8	198	0	0	0–1.8
2016	359	20	5.6	3.4–8.5	158	0	0	0–2.3
2017	NA	NA	NA	NA	158	0	0	0–2.3
2018	20	2	10.0	1.2–31.7	265	3	1.1	0.2–3.3
Total	986	194	19.7	17.2–22.3	2,297	21	0.91	0.5–1.4

NA: not applicable

**Table 2:** The host-specific occurrence of ruminant fascioliasis at MDVKK and SMTC.

Host species	MDVKK				SMTC			
	Total sample (*N*)	Total positive sample (*n*)	Occurrence of fascioliasis (%)	Confidence interval (95%)	Total animal slaughtered (*N*)	Total positive sample (*n*)	Occurrence of fascioliasis (%)	Confidence interval (95%)
Cattle	541	170	31.4	27.5–35.5	1128	19	1.7	1.0–2.6
Buffalo	219	19	8.7	5.3–13.2	245	2	0.8	0.1–2.9
Goat	221	5	2.3	0.7–5.2	924	0	0	0–0.4
Total	986	194	19.7	17.2–22.3	2,297	21	0.9	0.6–1.4

**Table 3:** The district-specific occurrence of ruminant fascioliasis at MDVKK.

Districts	Total sample (*N*)	Total positive sample (*n*)	Occurrence of fascioliasis (%)	Confidence interval (95%)
Papar	21	5	23.8	8.2–47.2
Kota Marudu	2	0	0	0–84.2
Tuaran	138	0	0	0–2.6
Penampang	40	0	0	0–8.8
Keningau	63	0	0	0–5.7
Tenom	2	0	0	0–84.2
Labuan	1	0	0	0–97.5
Bongawan	6	1	16.7	16.7–64.0
Lahad Datu	4	0	0	0–60.2
Ranau	424	160	37.7	33.1–42.5
Telupid	180	0	0	0–2.0
Sandakan	37	9	24.3	11.8–41.2
Sook	6	0	0	0–45.9
Tambunan	59	19	32.2	20.6–45.6
Kinabatangan	10	1	10	0.3–44.5
Total	986	194	19.7	17.2–22.3

## Data Availability

The data used to support the findings of this study are available from the corresponding author upon request, subject to permission from all authors and validity of the request.
